# Enumeration of Smallest Intervention Strategies in Genome-Scale Metabolic Networks

**DOI:** 10.1371/journal.pcbi.1003378

**Published:** 2014-01-02

**Authors:** Axel von Kamp, Steffen Klamt

**Affiliations:** Max Planck Institute for Dynamics of Complex Technical Systems, Magdeburg, Germany; The Pennsylvania State University, United States of America

## Abstract

One ultimate goal of metabolic network modeling is the rational redesign of biochemical networks to optimize the production of certain compounds by cellular systems. Although several constraint-based optimization techniques have been developed for this purpose, methods for systematic enumeration of intervention strategies in genome-scale metabolic networks are still lacking. In principle, Minimal Cut Sets (MCSs; inclusion-minimal combinations of reaction or gene deletions that lead to the fulfilment of a given intervention goal) provide an exhaustive enumeration approach. However, their disadvantage is the combinatorial explosion in larger networks and the requirement to compute first the elementary modes (EMs) which itself is impractical in genome-scale networks.

We present MCSEnumerator, a new method for effective enumeration of the smallest MCSs (with fewest interventions) in genome-scale metabolic network models. For this we combine two approaches, namely (i) the mapping of MCSs to EMs in a dual network, and (ii) a modified algorithm by which shortest EMs can be effectively determined in large networks. In this way, we can identify the smallest MCSs by calculating the shortest EMs in the dual network. Realistic application examples demonstrate that our algorithm is able to list thousands of the most efficient intervention strategies in genome-scale networks for various intervention problems. For instance, for the first time we could enumerate all synthetic lethals in *E.coli* with combinations of up to 5 reactions. We also applied the new algorithm exemplarily to compute strain designs for growth-coupled synthesis of different products (ethanol, fumarate, serine) by *E.coli*. We found numerous new engineering strategies partially requiring less knockouts and guaranteeing higher product yields (even without the assumption of optimal growth) than reported previously. The strength of the presented approach is that smallest intervention strategies can be quickly calculated and screened with neither network size nor the number of required interventions posing major challenges.

## Author Summary

Mathematical modeling has become an essential tool for investigating metabolic networks. One ultimate goal of metabolic network modeling is the rational redesign of biochemical networks to optimize the production of certain compounds by cellular systems. Accordingly, several optimization techniques have been proposed for this purpose. However, for large-scale networks, an effective method for systematic enumeration of the most efficient intervention strategies is still lacking. Herein we present MCSEnumerator, a new mathematical approach by which thousands of the smallest intervention strategies (with fewest targets) can be readily computed in large-scale metabolic models. Our approach is built upon an extended concept of Minimal Cut Sets, the latter being minimal (irreducible) combinations of reaction (or gene) deletions that will lead to the fulfilment of a given intervention goal. The strength of the presented approach is that smallest intervention strategies can be quickly calculated with neither network size nor the number of required interventions posing major challenges. Realistic application examples with *E.coli* demonstrate that our algorithm is able to list thousands of the most efficient intervention strategies in genome-scale networks for various intervention problems.

This is a *PLOS Computational Biology* Methods article.

## Introduction

Stoichiometric and constraint-based modeling techniques such as flux balance analysis or elementary modes analysis have become standard tools for the mathematical and computational investigation of metabolic networks [1]–[4]. Although these methods rely solely on the structure (stoichiometry) of metabolic networks and do not require extensive knowledge on mechanistic details, they enable the extraction of important functional properties of biochemical reaction networks and deliver various testable predictions. The steadily increasing number of reconstructed and examined genome-scale metabolic network models of diverse organisms proves that methods for constraint-based modeling can deal with networks comprising up to several thousands of metabolites and reactions [1].

Metabolic networks consisting of *m* internal metabolites and *n* reactions can be formalized by an *m×n* stoichiometric matrix **N**. A common assumption of constraint-based methods is that the network is in steady state (i.e., the metabolite concentrations do not change) resulting in a system of homogeneous linear equations
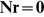
(1)where **r** is the vector of (net) reaction fluxes or reaction rates. In addition, the non-negativity constraints on fluxes through irreversible reactions must be fulfilled:
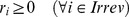
(2)(*Irrev* comprises the indices of the irreversible reactions). The two constraints (1) and (2) form a convex polyhedral cone (the *flux cone*) in the *n*-dimensional space of the rate vectors **r**. Flux Balance Analysis (FBA; [3]) searches for optimal flux distributions within this cone that maximize a given linear objective function
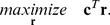
(3)Typical objective functions are maximization of growth (or biomass yield) or of the yield of a certain product. For FBA, the irreversibility constraint (2) can be refined to general upper and lower boundaries for each reaction rate *r_i_*:
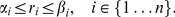
(4)Elementary-modes analysis [2], [5] is another stoichiometric technique facilitating the exploration of the space of feasible steady state flux distributions by means of particular flux vectors **e** fulfilling the basic constraints (1) and (2) and in addition a non-decomposability property. The latter demands that an elementary mode **e** is irreducible (or support-minimal), hence, there is no vector **r**≠**0** obeying (1) and (2) and
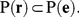
(5)Here, P(**r**) and P(**e**) represent the *support* of **r** and **e**, respectively, i.e., they contain the indices of the vector elements being non-zero: P(**t**) = {*i* | *t_i_*≠0}. Elementary modes (EMs) represent stoichiometrically balanced metabolic pathways or cycles and several important properties of a metabolic network can be analyzed by its unique set of EMs [2], [5]. EMs correspond to extreme rays of convex cones and can be computed as such [6], [7].

One ultimate goal of metabolic network modeling is the targeted manipulation of the network behavior. A typical application is metabolic engineering where one is interested in the optimization of the production of a certain compound by a given host organism. A number of constraint-based optimization techniques have been proposed for this purpose [2], [8], [9], [10], [11], [12]. FBA can directly be used to determine the optimal (maximal) value for a given optimization problem (e.g., maximal yield of biomass or of a certain chemical when growing on a certain substrate). This approach, however, cannot yet explain which manipulations will eventually drive the cell towards this optimum. A simple approach would be to use flux-variability analysis (FVA, [13]) to analyze how the feasible ranges of stationary fluxes in a metabolic network would change when switching from the wild-type to a desired phenotype. More sophisticated and directed FBA-based optimization routines operate on the principle put forward by the OptKnock approach [8]. Here, the key idea is to search for interventions that lead to obligatory coupling between the production of biomass and of a desired compound. Mathematically, OptKnock is a bilevel optimization problem where the inner problem defines biomass optimization as the cellular objective and where the outer optimization problem is to search for reaction removals (represented by integer variables) that lead, under consideration of the inner problem, to maximal product formation. The bi-level optimization coupling can be reformulated as a single level mixed integer linear program (MILP). Successful applications (e.g. [14]) and several refined variants of OptKnock (including, for example, RobustKnock [9] and OptORF [11]) have been published (for a review see [12]). The advantage of FBA-based approaches is that they can readily be applied to genome-scale networks. However, a potential disadvantage is that they deliver particular solutions only where often multiple alternate solutions exist which might be equally or even more relevant than the found solutions. Some methods have therefore been proposed to enumerate intervention strategies. A brute-force approach would be to test all single, double, triple … reaction knockouts with respect to their impact on the objective function [15], [16]. Suthers et al. [15] used this method to enumerate synthetically lethal reaction sets and found that this search becomes prohibitive in genome-scale networks for interventions with more than two or three reaction knockouts (the upper limit set in [16] was also three). They designed therefore a more directed search algorithm based on a bi-level optimization method formulated as a mixed integer linear program (MILP) [15]. However, to the best of our knowledge, enumerated knockout sets in genome-scale networks did not exceed a cardinality of three. This is a serious limitation because complex interventions problems may require 5, 6, 7 or more knockouts, even in medium-scale networks (see [17] and the examples in the Results section).

The method of Minimal Cut Sets (MCSs) directly addresses the enumeration of metabolic intervention strategies [10], [18], [19]. MCSs specify minimal sets of reactions whose removal (knockout) will block certain undesired (target) flux distributions. For example, one can compute (i) MCSs that block growth; (ii) MCSs that disable the production of a certain compound; (iii) MCSs that block all flux vectors where a certain compound is produced with a low (including zero) yield. In the context of MCSs, the term “minimal” refers to the property that reaction cuts specified by any proper subset of an MCS are insufficient to ensure the full repression of the undesired behaviour. In this regard, the minimality of MCSs is similar to the minimality or non-decomposability property of elementary modes specified by equation (5). In fact, there is a dual relationship between MCSs and EMs: the MCSs blocking a certain set of target flux vectors are the *minimal hitting sets* of the set of (target) EMs that generate these behaviors [19], [20]. By this property, each MCSs must hit (knockout) at least one utilized reaction from each EM. As a consequence, MCSs can be computed as minimal hitting sets (or so-called hypergraph transversals) of the target modes, for instance, by the Berge algorithm (see [20]) or by Binary Linear Programming [21].

Another approach to compute MCSs, which exploits the inherent dual relationship between EMs and MCSs, was recently presented by Ballerstein et al. [22]. Briefly, the MCSs of a given metabolic network can be computed as certain EMs of a dual network; the latter being derived by a simple transformation of the (primal) network. This finding makes it possible to calculate MCSs by using optimized algorithms for EM computation [7].

However, there are two potential problems related to MCSs. First, when the reactions contained in an MCS are removed, we are sure that the targeted network functions are disabled but other (desired) functions might be blocked as well. For instance, it can occur that an MCS which disables low-yield pathways synthesizing a desired product also blocks growth of the organism making this MCS impractical. To prevent such side effects, the concept of *constrained minimal cut sets* (cMCSs) was introduced by Hädicke and Klamt [10] where not only undesired but also desired functionalities (to be preserved) can be specified. When the EMs are available, an adapted Berge algorithm can be used to conveniently compute cMCSs by specifying in addition to the target modes (expressing the unwanted behaviour) a set of desired modes expressing the functionality that must be preserved. A cMCS will hit all target EMs and keep a (user-specified) minimal number of desired EMs. As shown in [10], cMCS provide a very flexible and powerful approach to enumerate intervention strategies; many other techniques such as Minimal Metabolic Functionality [2], [17], and the aforementioned OptKnock and RobustKnock may be reformulated as special cMCSs problems. cMCSs are also well-suited to identify knockout combinations leading to coupled growth and product formation.

The second and more serious problem of (c)MCSs is that their full enumeration in large/genome-scale networks becomes prohibitive. The algorithms requiring as inputs the target (and possibly desired) EMs are usually not applicable: despite large progress in algorithmic design [7] the full set of EMs is often not computable at genome-scale. For the same reason, the dual approach of Ballerstein et al. [22] cannot be applied either.

On the other hand, for the purpose of applying MCSs in real networks, those with the smallest number of elements are usually the most relevant. Thus, it is worthwhile to consider computing only the (c)MCSs with low cardinality. The effective enumeration of the smallest cut sets is therefore the key goal of the present work.

Usually, the unwanted/desired functionalities to be disabled/kept in a metabolic network can be described by sets of linear equalities and inequalities over the fluxes. For the purpose of computing MCSs, we could therefore use an exhaustive FBA-based scheme by testing all single, double, triple and higher knockout sets whether they are suitable cut sets or not. The formulation of FBA problems would circumvent the problem to enumerate the EMs first. However, as discussed above, this approach becomes problematic if larger knockout sets are required to solve an intervention problem, as it must test a large number of candidate sets with increasing MCS size (the number of candidates grows with 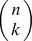 where *n* is the number of possible cuts and *k* the size of cut set candidates). Therefore, it is not normally possible to find genome-scale MCSs in reasonable time with more than 4 knockouts using this scheme.

Whereas the direct calculation of smallest MCSs in large-scale networks cannot be properly addressed yet by current methods, a method for computing the smallest (or shortest) EMs in genome-scale networks was recently presented by de Figueiredo et al. [23]. This algorithm formulates the search for the EMs with fewest elements as a Mixed Integer Linear Programming (MILP) problem and delivers in the *k*-th iteration the *k*-th shortest EM (hence, it starts with shortest EM, delivers then the second shortest and so forth). As shown by the authors, this approach can readily be applied to genome-scale networks to find the first hundred or even thousand shortest EMs involving the fewest number of reactions.

The goal of the present work is to realize a similar approach for computing the *k*-smallest MCSs from a given network structure. We show that this can be achieved in two steps. First, the original network and the actual intervention goal are converted to its dual representation using the approach of Ballerstein et al. [22]. We then compute the shortest EMs (up to a certain size or number) in the dual network by employing a modified algorithm of de Figueiredo et al. [23]. As the EMs in the dual network correspond to the MCSs of the primal, the shortest EMs in the dual system will represent the smallest MCSs of the original network.

The paper is organized as follows: we will first briefly review the approach of de Figueiredo et al. for computing *k*-shortest EMs and introduce several modifications improving the performance of this algorithm. In particular, we will make use of certain features of MILP solvers provided for effective enumeration of solutions of a MILP problem.

Thereafter we will describe how the network constraints (including inhomogeneous constraints) and the intervention goal have to be translated into their dual description in which we can then enumerate the shortest EMs to obtain the smallest MCSs in the primal network. We shall also explain how *constrained* MCSs can be computed within this framework. Finally, to demonstrate the power of our new approach we will exemplify its use by computing relevant intervention strategies (of different complexities) in iAF1260, a genome-scale metabolic model for *E.coli* [24]. These benchmarks demonstrate, for example, that our approach enables us to enumerate synthetic lethals of *E.coli* up to size 5 which was not possible before. Moreover, we show that the algorithm facilitates the calculation of thousands of the minimal intervention strategies that lead to growth-coupled synthesis of certain compounds by *E. coli*.

For the sake of simplicity, throughout the manuscript we will deal with reaction cut (or knockout) sets, which must in practice be translated to gene knockout sets to construct the corresponding mutants. This transformation can be easily achieved if the corresponding gene-enzyme-reaction associations are available. The latter could also directly be included in the problem formulations given below to compute gene (instead of reaction) cut sets.

## Methods

### MILP framework for enumerating shortest elementary modes

#### Representing sets in a MILP problem

Both elementary modes and minimal cut sets can be represented as sets of reactions (sets of active reactions in case of EMs and sets of deleted reactions in case of MCSs). Since we are mainly interested in the composition and size (cardinality) of EMs and MCSs it is important to represent them efficiently in the MILP problem to be formulated. Here we will make use of *indicator variables*, a feature provided by advanced MILP solvers (such as CPLEX – we will refer to this solver throughout the paper but most of the used functionality is also available in other MILP solvers). An indicator is a binary variable that can be thought of as controlling the activity of one or more constraints. Indicators can be part of the objective function and constraints controlled by indicators may in turn influence other indicator variables as well. An important application of these variables that we use here is to indicate whether another variable is equal or greater than zero. More precisely, we use an indicator variable *z_i_* to indicate whether a real and non-negative variable *x_i_* is greater than zero:
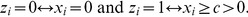
(6)Importantly, in the following we can use *c* = 1 as threshold for a variable to be greater than zero because the solutions (the EMs) to the MILPs set up here are scalable by arbitrary factors due to the unboundedness of the *x_i_*. In principle, a different positive value could be chosen for *c* but setting *c* = 1 can be expected to not cause any particular numerical problems.

The functionality of indicator variables is often implemented by a “big M” formulation with integer variables (cf. eqs.(1) and (2) in [23]), but as this can lead to numerical problems, the use of indicators is now preferred (note that by constraints such as (6) indicator variables are more powerful than simple binary integer variables). For reasons of clarity, we directly use indicator variables in the formulations of MILPs where needed (in CPLEX they are set up through API functions) and leave their integration with the regular MILP constraints to the MILP solver. The use of binary indicator variables *z_i_* will turn the linear problems discussed so far into a MILP problem with discrete and continuous variables. In contrast to Figueiredo et al. [23], we do not demand that the coefficients in the stoichiometric matrix are integers; in the latter case one would obtain a pure integer linear problem.

#### MILP formulation for computing the shortest EM

We now rephrase the MILP problem presented in [23] for determining the shortest EM but here with explicit use of indicator variables. In the following, we assume that the network and the stoichiometric matrix **N** contain only irreversible reactions. This is no limitation since reversible reactions can be decomposed into two irreversible ones, one in forward and one in backward direction. Using (6) we now define indicator variables for the reaction rates *r_i_*:
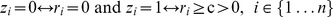
(7)(as mentioned above we can safely use *c* = 1). For all reaction pairs (*s,t*), *s,t* ∈ {1,…, *n*} that were derived from the same formerly reversible reaction we demand that only one of both (directions) can be active to avoid spurious cycles:
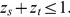
(8)To exclude the trivial vector **r** = **0**, we demand in addition that an EM must contain at least one active reaction:
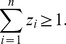
(9)The determination of the shortest EM can then be expressed as an optimization problem with the objective function
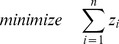
(10)subject to the linear constraints (1), (2) and the integer constraints (7)–(9). A solution to the system above assigns values to the vectors **r** and **z** so that all constraints are fulfilled and the value of the objective function is minimized. The vectors **r** and **z** contain the shortest EM in vector and set representation, respectively. Note that this MILP does not yet contain constraints enforcing the elementarity of the solution. However, due to the non-decomposability property of EMs all solutions minimizing (10) must be EMs and, moreover, they are the shortest EMs as they involve a minimal number of reactions.

#### Standard MILP formulation for enumerating shortest EMs

The actual enumeration of the *k*-shortest EMs as implemented by de Figueiredo et al. [23] starts with the shortest EM and iteratively yields new EMs of increasing size. Because typically many EMs of the same size exist all EMs of a given size are returned before larger ones are found. As an essential step, EMs found in previous iterations need to be excluded by proper constraints on the *z_i_* (see below). The pseudo-code for enumerating the *k* shortest EMs thus reads:

**ALGO1: k-shortest EMs via de Figueiredo et al. [23]**

ems = {};

k = 0;

WHILE k < MaxNumEM

   *k++;*

   newem = solveMILP( ); /* calculate one optimal solution

(k-shortest EM) of the MILP */

   *ems = ems ⋃ {newem};*

   *add_exclusion_constraint(newem);*

ENDWHILE

The last step in the loop remains to be explained, the addition of exclusion constraints to the MILP which make sure that duplicates or supersets of already identified EMs will not be returned as solutions by subsequent *solveMILP()* calls. An exclusion constraint takes the following form (cf. eq. (7) in [23]): Let 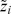
∈{0,1} be the value of *z_i_* in the MILP solution for the EM *newem*. The constraint added for this EM reads:
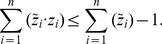
(11)This constraint (also known as integer cut) makes sure that solutions found by the next optimizations cannot contain the complete set of reactions used in the current EM thus excluding also supersets of the current EM from the solution space. Similar constraints have frequently been used also in other metabolic network studies when searching for multiple solutions of a given optimization problem (see, e.g., [15]). Once an exclusion constraint has been added to the system it has to be kept for all further iterations. Consequently, the number of constraints in the MILP continuously increases.

#### Enumeration of EMs with fixed size

In this subsection, we propose a modified scheme for enumeration of shortest EMs. We first introduce an additional size control constraint
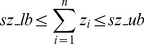
(12)(with *sz_lb, sz_ub* ∈ℕ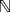) specifying how many elements an EM may contain.

We restate that the exclusion constraints (11) are needed to prevent supersets of known EMs from being erroneously identified as EMs. If all EMs up to size *d* are known and exclusion constraints for them have been added, then the next solution will be an elementary set of size *s*>*d* (unless all sets have already been found in which case the MILP will be infeasible). We can therefore fix the size control constraint to *s* (*sz_lb* = *sz_ub* = *s*; normally starting with *s = d+1*) so that only EMs of exactly this size are calculated. As long as solutions of size *s* are enumerated, exclusion constraints for the solutions would only be required to prevent the same solution from being found again because supersets of these solutions are already excluded by the size control constraint. This opens up the possibility to use the MILP without adding exclusion constraints at all while generating the solutions of a fixed size *s* by using the warm start feature of advanced MILP solvers. With this feature, the preprocessing will not be reiterated when computing the EMs of size *s* and the search tree generated during the previous search for a solution can be reused for finding the next solution. This improves the efficiency of the whole procedure and by continuing with the same search tree it is also made sure that the same solution is not returned twice. In CPLEX, this feature is provided by a function *populate* allowing the enumeration of all possible solutions to a MILP problem. Also, when searching for EMs of a fixed size only, the problem does not require an optimization over the sum of the *z_i_* any more (since the latter is fixed as a constraint) and becomes thus merely a search for a feasible solution which is potentially easier to solve.

Importantly, when all EMs of size *s* have been, enumerated exclusion constraints for the found EMs must be added as usual before continuing with *s+1* (to avoid that supersets of EMs of size *s* will be found in subsequent iterations). However, the advantage of this approach remains because exclusion constraints need not be added when processing all EMs with cardinality *s*. We summarize the basic scheme as follows:

**ALGO2: k-shortest EMs via fixed size**

newem = solveMILP( ); /* calculate first optimal solution of the

             MILP (the shortest EM) */

s = objective_value(newem); /* s: current lower boundary of EM

              size */

ems = {newem};

remove_objective_function( ); /* remove the minimization over the z_i_ */

WHILE s< = maxEMsize

 setConstraint(EMsize = s); /* fixed size of EM */

 newems = populateMILP( ); /* enumerate all feasible EMs

                with size s */

 *ems = ems ∪ newems;*

 *add_exclusion_constraints(newems);*

 *s++;*

ENDWHILE

The search that is conducted during a *populateMILP* call can usually be halted (e.g., by a time limit) and continued so that the solutions found so far can be accessed before the search is finished. This means that even if for a given set size more solutions than can be practically calculated exist it is still possible to use this scheme to get at least a partial result.

### Enumeration of smallest MCSs by enumeration of shortest EMs in the dual network

We present now the key methodological development of this work showing that the basic algorithm for enumerating shortest EMs introduced in the previous section can also be used to compute smallest MCSs. The procedure is based on the duality properties of EMs and MCSs presented by Ballerstein et al. [22] which we outline in the following. A necessary first step to establish the scheme is to describe the undesired network functionality (the “target flux vectors” **r** to be disabled by the MCSs) by a suitable inequality constraint
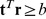
(13)where **t** is a (*n*×1) vector. Usually, **t** corresponds to a single row with zeros except a single 1 for a target reaction (rate) whose operation is to be blocked (e.g. biomass formation if we searched for synthetic lethals). Setting in addition *b* to 1 we would target all flux vectors in which the rate of the target reaction is non-zero (in our context we can again set *b* to an arbitrary value greater than zero without loss of generality).

Constraint (13) specifying the target flux vectors can be generalized to:
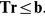
(14)Here, matrix **T** (of size *t*×*r*) together with 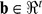
poses *t* inhomogeneous inequality constraints defining the *target flux polyhedron* (which may be bounded becoming then a polytope). It must be made sure that the zero flux vector is not contained in the target flux polyhedron as it can not be blocked by reaction knockouts. A nice feature of (14) is that we may directly include inhomogeneous constraints to characterize target flux vectors (with maintenance ATP demand as a typical example).

In addition to (14) and to the standard network constraints (1) and (2), Ballerstein et al. augmented the system by equality constraints setting all reaction rates to zero
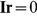
(15)(**I** is the (*n*×*n*) identity matrix). These constraints ensure that the system becomes infeasible as the zero flux vector implied by (15) contradicts (14). Note that (15) can be seen as the maximal (trivial) cut set knocking out every reaction in the network. In fact, the MCSs correspond to minimal subsets of the homogeneous equations in (15) which ensure (induce) inconsistency of the inequality system posed by constraints (1), (2), (14) and (15). Minimal subsets of constraints that induce inconsistency of an inequality system are also known as *irreducible inconsistent subsets* (IISs; [25]). Generally, IISs can be calculated as follows: using the Farkas Lemma, the infeasible primal system is converted to its dual system which is ensured to be consistent. It can be shown that the IISs of the primal system correspond to extreme rays (and thus EMs) in the dual system which makes it possible to calculate them using methods from EM computation. Since IISs in our particular case may, in general, also contain constraints from (1) or (2), a modified algorithm was introduced in [22] to ensure that only those IISs ( = EMs in the dual system) are computed which are minimal with respect to the constraints in (15) and correspond thus to the MCSs.

We thus need to transform the primal system defined by (1), (2), (14), (15) into its dual which can be written as follows (cf. equation (8) in [22]; **N***_dual_* is the “dual stoichiometric matrix” and **r***_dual_* the dual “rate” vector):
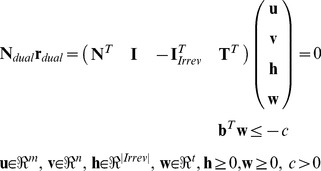
(16)The (sub-)matrix 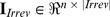 contains the identity matrix for irreversible reactions of the primal system and is filled with *n*-|*Irrev*| zero rows at the position of reversible reactions (note that reversible reactions of the primal system need not to be split before dualizing the system; however, reversible reactions affected by (14) must sometimes be split to properly describe the target flux polyhedron). As described above, the MCSs in the primal correspond to particular EMs of the dual system (16) which have minimal support in the **v** variables. The dual variables *v_i_*, *i*∈{1 … *n*} are thus of particular importance as their values indicate which reactions participate in an MCS. Concretely, if *v_i_*≠0 then reaction *i* is part of the MCS (irrespective of the sign of *v_i_*), if *v_i_* = 0 then it is not. Therefore, similar as we did for reversible reactions when computing shortest EMs, both positive *and* negative values of *v_i_* must be checked with indicators and in order to facilitate this each *v_i_* is split into two variables, *vp_i_* and *vn_i_*, both with the lower bound 0. Furthermore, since **h**≥**0** and because the MILP can directly operate on inequalities, we can rewrite (16) to:
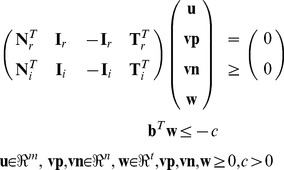
(17)(the sub-matrices with subscript *i* refer to the part of the irreversible reactions and subscript *r* to the part of the reversible reactions of the primal system). As mentioned above, for the *vp_i_* and *vn_i_* we introduce the associated indicators *zp_i_* and *zn_i_*, and (in equivalence to (8)) the constraints
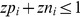
(18)stating that *vp_i_* and *vn_i_* cannot be active simultaneously. The constant *c* in (17) can again be set to any positive value (e.g., to 1); this will not change the set of minimal non-zero combinations of *vp_i_* and *vn_i_* fulfilling (17) which are relevant for the optimization problem formulated below (eq. (19)).

After dualization, we can now compute the smallest MCSs of the primal system by applying algorithm ALGO2 in the dual system. As constraints we need to consider (17) (replacing (1) and (2) from the primal system) as well as (18) and as objective function we exchange (10) with
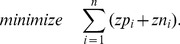
(19)Furthermore, because the presence of one reaction in a concrete solution is now indicated by two *separate* variables, the exclusion constraints (11) must be adapted accordingly to (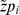 *and* 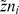 are the values of a given concrete solution and 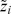 is a shortcut for 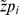
*+*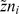):
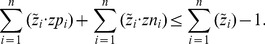
(20)In this way both positive and negative values of the original *v_i_* are counted in the same way towards reaction participation in the MCS. Finally, for the same reason, the size control constraint (12) sums here over *zp_i_*+*zn_i_* as in the objective function (19).

The MCSs of the primal network are eventually obtained by taking the **z**-vector of the solutions found in the dual; **z** is obtained by collapsing *zp_i_* and *zn_i_*: *z_i_* = *zp_i_*+*zn_i_*.

### Enumeration of smallest constrained MCSs

In the previous subsection we dealt with enumeration of smallest MCSs, however, we have not yet clarified how *constrained* MCSs can be computed by this approach. As it turns out, this is straightforward: one first enumerates the smallest MCSs blocking the undesired flux vectors as described above. We can assume that the desired flux vectors (of which at least one has to be kept) is formulated by appropriate inequalities - similar as for the targeted undesired flux vectors in (14):
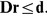
(21)We can then filter the true cMCS from the set of (unconstrained) MCSs by testing for each MCS with a separate linear program whether the removal of the reactions in the MCS still allows the network to perform the desired function, i.e., whether the system given by (1), (2), and (21) is feasible when setting the rates of the reactions contained in the MCS to zero. From our experience, the computational costs for these tests are negligible compared to the calculation of the smallest MCSs, even if hundred thousand MCSs have to be tested (see Results section).

### Implementation

The MCSEnumerator method has been integrated as a new functionality in the *CellNetAnalyzer* package, a MATLAB toolbox for biological network analysis [26], [27]. The implementation uses the IBM ILOG CPLEX Optimization Studio V12.4 for solving the respective MILP and LP problems. Arbitrary intervention problems can be defined by providing the respective matrices and vectors describing the network and the desired and undesired flux vectors. The resulting MILPs are set up via the JAVA-CPLEX API and MATLAB's integrated JVM while for running the LPs the MATLAB-CPLEX interface is used. A separate package containing the data and script files needed for running the iAF1260 examples discussed herein can be downloaded from http://www.mpi-magdeburg.mpg.de/projects/cna/etcdownloads.html.

## Results

We analyze basic properties of the runtime behavior of our algorithm by means of three realistic benchmark problems with different complexities. All computations were performed with the CPLEX 12.4 MILP solver. When using multiple threads deterministic parallel mode was used to get repeatable behaviour. The search tree that CPLEX dynamically constructs took up less than 3 GB of RAM for all the systems used here.

### Enumeration of MCSs blocking growth in a model of the central *E. coli* metabolism

In order to compare our MILP-based MCS enumeration scheme to other approaches the same benchmark problems as in Table 1 in [22] were used. The target of the (unconstrained) MCSs in these problems is the deactivation of biomass synthesis in a smaller model of the central metabolism of *E. coli* for growth under different substrates (acetate, succinate, glycerol, glucose). The MCSs determined in this way will thus correspond to the synthetic (reaction) lethals for *E. coli* (whose compositions depend strongly on the provided substrate). Before using the different MCS calculation routines the metabolic network is compressed by combining correlated reactions (operating with a fixed ratio under steady state conditions) to single cumulated reactions [6]. The compression in the primal system can also conducted if the computation is done in the dual system. MCSs found in the compressed network must be decompressed after calculation [18].

**Table 1:** Enumeration of all MCSs (synthetic reaction lethals) in a medium-scale metabolic model of *E. coli*.

Problem				MCSs via EMs from original network	MCSs as EMs of the dual system		
substrate	threads	size limit	MCS number	Compute first EFMs, then minimal hitting sets	Method of Ballerstein et al. [22]	ALGO1: Iterative MILP	ALGO2: MILP populate with fixed EM sizes
acetate	1	-	309	0.6 s	0.4 s	19 s	3 s
succinate	1	-	1623	6.4 s	7.0 s	499 s	32 s
glycerol	1	-	3733	36.8 s	37.4 s	61.4 min	3.5 min
glucose	1	-	4960	181.3 s	188.7 s	356.6 min	21.2 min
glucose	1	4	423	43.9 s	not possible	92.5 s	4.2 s
glucose	4	-	4960	unsupported	unsupported	228 min (698.0 min)	18.5 min (56.8 min)
glucose	12	-	4960	unsupported	unsupported	62.9 m (633.4 m)	5.6 min (58.3 min)

Computation times for MCSs that disable growth in an *E. coli* metabolic network model of the central metabolism under different substrate uptake conditions (cf. Table 1 in [22]). The full/compressed networks contain 89/25 metabolites and 106/42 reactions. Conversion of MCSs in the compressed network to those in the full network takes negligible computation time for the cases shown here. For iterative solve (ALGO1) CPLEX dynamic search was used while for populate calls (ALGO2) traditional branch-and-cut was applied. In the fifth problem, only the MCSs up to size 4 were calculated. The computation times for the classical approach (EM+minimal hitting sets) and for the dual approach of Ballerstein et al. in the first four problems are the same as in [22]; note that neither method currently supports multiple threads. For calculations using multiple threads the physical computation time is shown with the sum of computation times (CPU times) over all threads in brackets. The calculations with 1 and 4 threads were performed with an Intel Q9550 desktop processor (4 cores) while for 12 threads a cluster node with two Intel Xeon DP X5650 processors (each 6 cores) was used.

The number of calculated MCSs and computation times are shown in Table 1. As a first observation, it is apparent that calculation of EMs followed by the Berge algorithm (computing MCSs as the minimal hitting sets of the selected target EMs; Haus et al. 2008) is the most efficient of the shown MCSs calculation methods. The approach of Ballerstein et al. to compute primal MCSs as EMs in the dual system performs similar to EM calculation+Berge algorithm in the (primal) network but in its current implementation it requires a lot of memory. For this reason, the MCSs for glucose could not exhaustively be enumerated by this approach on the computer used (with an effective memory limit of 2GB per process).

Although the MILP algorithm developed herein was actually developed to compute the *smallest* MCSs, we can use it here even for enumerating *all* of them. The EMs in the dual network (the MCSs in the primal) where computed with both MILP formulations for shortest EM calculation: ALGO1 (the original approach by de Figueiredo et al. [23] implemented with indicator variables) and the ALGO2 approach calling the *populate* sub-routine for fixed EM sizes. Generally, applying the MILP formulations to the dual system is at first sight comparatively slow even when using multiple threads. Nonetheless, it is apparent that solving the dual system with our new ALGO2 is more efficient (∼17 times faster) than ALGO1 based on the scheme used by de Figueiredo et al. [23]. As can be seen for the MCSs with glucose as substrate, increasing the number of threads from 1 to 4 on the same CPU decreases the time needed for computation to some extent when using ALGO1 or ALGO2. Using 12 threads on a compute cluster node yields a more noticeable speed improvement but, as in the case of 4 threads, the combined computation times of all threads is still larger than in the case where a single thread is used.

The main advantage of our new approach can be seen in the case where only the MCSs up to size 4 have to be calculated (fifth row in Table 1): here the dual approach in combination with ALGO2 is clearly the fastest way to determine small MCSs among the approaches compared.

### Enumerating synthetic lethals in an *E. coli* genome-scale network

As described in the Introduction section, the direct calculation of EMs and MCSs in genome-scale networks is normally infeasible. For this reason, the Berge algorithm and the dual system approach by Ballerstein et al. used in the previous example become impractical. In contrast, with the MILP approach enumerating shortest EMs in the dual system as proposed here, calculation of small MCSs becomes possible.

To demonstrate this, we use the *E. coli* genome-scale network iAF1260 [24] that accounts for 1260 ORFs and defines the reversibilities of the included reactions. In total, this network comprises 1668 internal metabolites and 2382 reactions including 304 exchange reactions with the environment and 29 spontaneous reactions. The intervention goal for the MCSs to be computed is again to disable growth (biomass formation) when glucose is available as sole carbon source. The glucose uptake rate was fixed to 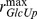 = 10 *mmol/(gDW⋅h)* and the ATP maintenance requirement was set to the standard value of 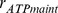 = 8.39 *mmol/(gDW⋅h)*. Analogous to Suthers et al. [15] we considered a cell viable if it has a growth rate larger than *μ^min^*≥0.01⋅*μ^max^* = 0.0093 *h^−1^*. With these inhomogeneous conditions, the MCSs will thus correspond to synthetic reaction lethals as also computed by Suthers et al. [15], where full enumeration for MCSs up to size 3 was achieved (some MCSs of size 4 could also be determined). With glucose and oxygen available 152 reactions are disabled as suggested by the gene-regulatory model included in the iAF1260 reconstruction. A subsequent flux variability analysis revealed 991 blocked reactions in total and these were removed from the network. In addition, spontaneous and exchange reactions, of which 23 resp. 97 remain after removing blocked reactions, were not allowed to take part in any MCS. After removing the blocked reactions network compression by combining correlated reaction sets was again applied by which the (primal) network could be reduced to 562 metabolites and 936 (lumped) reaction subsets of which 816 can be knocked out. By using ALGO2 in the dualized system, for the first time it was possible to fully enumerate all synthetic (reaction) lethals of sizes 1 to 5 as shown in Table 2 yielding a total set of 2486 MCSs. Although the last iteration (MCSs with 5 knockouts) took several days all of them could be determined. Comparison of the runtimes of our MCSEnumerator implementation and of SL Finder (used in [15]) for the calculation of MCSs of size two and three indicates that our algorithm is more than 100 times faster therefore allowing full enumeration of synthetic reaction lethals also of size 4 and 5.

**Table 2:** Enumeration of smallest MCSs (synthetic reaction lethals) disabling growth in a genome-scale network model of *E.coli*.

MCS size	number of MCSs	physical time with MCSEnumerator	physical time with SL Finder
1	277	11.1 s	[included]
2	96	39.1 s	91 min
3	247	16.8 min	>75.5 h *)
4	402	18.5 h	n/a
5	1464	410.4 h	n/a

MCSs (synthetic reaction lethals) that disable growth in an *E. coli* genome-scale metabolic network with glucose as sole carbon source. The full/compressed networks contain 1668/562 metabolites and 2382/936 reactions. For computation 12 threads on a cluster node with two Intel Xeon DP X5650 processors (each 6 cores) were used. The computation time given in each row specifies the time needed to calculate the MCSs of the respective size. The total computation time for MCSs of size 1–5 was thus ∼430 h. In order to get comparable run times the SL Finder was executed on the same computer with GAMS 24.1.3 (using CPLEX 12.5.1 as solver). All physical memory was made available and up to 9 GB were used during optimization. The MCSEnumerator calculations were also done on a typical desktop PC with a quad-core CPU (Intel(R) Core(TM) i5-3570, 3.40 GHz) showing that the computation times increase only moderately by approximately 50%.

*) Only 226 synthetic triple lethals (which are all contained in the MCSs found by MCSEnumerator) could be calculated after which optimization could not be continued due to numerical problems reported by the solver.

We also tested the homogeneous version of the above intervention problem, that is, we calculated the MCSs blocking growth without the additional constraints for ATP maintenance (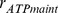 is a free flux), without restriction on glucose uptake and without the minimum threshold for the growth rate (all flux vectors with biomass production >0 have thus to be blocked). As expected, for we found less MCSs of size 1–5 (1933 in total) because the target polyhedron containing the target flux vectors was expanded leading to larger MCSs with more than 5 reaction deletions. We also observed that the computation of the MCSs in the homogeneous problem was much faster (∼17 hours) than for the inhomogeneous scenario (∼430 hours) indicating that inhomogeneous constraints may complicate the whole calculation procedure.

### Constrained MCSs for coupling anaerobic growth and ethanol production in *E. coli*

The following third example relates to a typical problem of finding rational intervention strategies for metabolic engineering purposes. We here focus on a biotechnologically relevant application, namely to let *E. coli* produce a biofuel (ethanol) from glucose. The intervention goal is thus to disable flux vectors with a low ethanol yield in *E. coli* (undesired behavior) while retaining the capability for both maintenance and growth of the bacterium under anaerobic conditions (desired functionality). This forms a constrained MCS problem. All cMCSs that fulfill the stated requirements will lead to obligatory coupling between growth and ethanol formation.

We used again the iAF1260 genome-scale network model of *E. coli* metabolism but this time with the oxygen uptake removed to establish anaerobic conditions on the network. As before, glucose is the only available carbon source. To study the effect of different capacities for substrate uptake, we considered two possible limits for the glucose uptake rate: 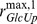 = 10 *mmol/(gDW⋅h)* and 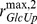 = 18.5 *mmol/(gDW⋅h)*. The latter value has been measured under anaerobic conditions where *E. coli* tends to exhibit higher substrate uptake rates [28]. The ATP maintenance requirement was set to 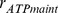
≥8.39 *mmol/(gDW⋅h)*.

With these values in mind, we formulated the following intervention goal: the task is to identify cMCSs that guarantee a minimal ethanol yield of 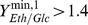 or, in a second scenario, of 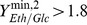. In addition, a minimum growth rate of at least *μ^min^*≥0.001 *h^−1^* was demanded.

With these inhomogeneous constraints we can now specify the target flux polyhedron containing all undesired network behaviors to be eliminated by the cMCSs:
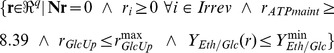
(22)(*Y_Eth/Glc_*(**r**) denotes the ethanol yield of the reaction rate vector **r**). The set of desired behaviors from which we want to keep at least some flux vectors is given by:
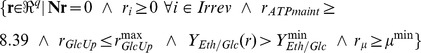
(23)(The constraints due to anaerobic growth (e.g., oxygen uptake is zero) were not restated in (22) and (23).)

With these values, several linear programs were run in a preprocessing step to explore network capabilities. For 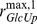 and 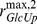, the maximal ethanol yield is 2 (molecules ethanol per molecule glucose). The maximum growth rate is 0.1955 *h^−1^* (for 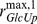) and 0.4954 *h^−1^* (for 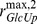) if we want to achieve an ethanol yield of at least 1.4 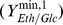; these values drop to 0.1356 *h^−1^*and 0.4827 *h^−1^*, respectively, for a minimal ethanol yield of 1.8 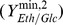. Hence, we can be sure that the set of desired behaviors is not empty.

We then computed the cMCSs. As described in the Methods section, the calculation of cMCSs (accounting for undesired *and* desired behavior) based on our approach requires to first compute the MCSs blocking the undesired behavior and to keep afterwards only those MCSs that admit the desired behavior. This test is done for each found MCS by solving a separate linear program (LP) which verifies whether the remaining network supports the desired behavior. To reduce the search space, blocked reactions for the network under desired ethanol production conditions were determined and removed in a preprocessing step using flux variability analysis [13]. In addition, 104 reactions were disabled for growth on glucose as suggested by the gene-regulatory model included in the iAF1260 reconstruction. The FVA then identifies 996 blocked reactions in total, which are removed from the network. Furthermore, the remaining 19 spontaneous and 94 exchange reactions were again not allowed to take part in the MCSs. The latter can be easily achieved by setting the upper bounds of the corresponding *zp_i_* and *zn_i_* indicator variables to zero.

After network compression, the (primal) network could be reduced to 562 metabolites and 958 (lumped) reaction subsets of which 845 can be knocked out.

Note also that the disruption of glucose uptake or ATP maintenance are valid MCSs deleting all undesired behaviors but they violate for trivial reasons the desired functionality (growth not possible) and can thus not be contained in any valid cMCSs. Such reactions being essential for the desired flux space could also be identified at an early stage and then be excluded from the search space.

Table 3 shows the results for the computation of the (c)MCSs for this problem. As we considered two different maximal glucose uptake rates and two different minimal ethanol yields we obtained four scenarios. We were able to enumerate all cMCSs up to size 7 in all four scenarios within 21 hours. For each scenario, after calculating first the (unconstrained) MCSs up to size 7, each MCS was tested with a LP whether the solution space of (23) is non-empty (i.e., whether the MCS is a valid cMCS). These tests took less than 7 minutes running time (single-threaded; on the same computer that was used for MCS calculation) for each of the four scenarios. Hence, the LPs account only for a negligible part of the overall computational costs.

**Table 3:** Computation of constrained MCSs leading to coupled ethanol and biomass formation by *E. coli* under anaerobic growth on glucose.

Scen.	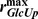	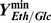	# MCSs	# cMCSs	cMCSs size					runtime [h] phys./CPU
					3	4	5	6	7	
1	10	1.4	185302	8342	8	46	283	1309	6696	20.5/207.6
2	10	1.8	153338	1987	0	0	77	317	1593	13.8/136.8
3	18.5	1.4	156477	8819	2	98	533	1737	6449	16.6/166.4
4	18.5	1.8	138675	4618	2	70	509	917	3120	20.9/212.2

Constrained MCSs up to size 7 that lead to ethanol synthesis with high yield in *E.coli* while slow growth is possible. Four scenarios were considered differing in the maximal glucose uptake rate (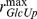; given in *mmol/(gDW⋅h)*) or/and in the demanded minimal ethanol yield (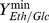; given in molecules ethanol per molecules glucose) in the strain to be constructed. The total number of MCSs (#MCSs) refers to knock-out sets blocking flux vectors with low ethanol yield; the number of constrained MCSs (#cMCSs) refers to the subset of MCSs which allow in addition growth above the minimum threshold (for details see text). For the cMCSs found, the distribution over cut set sizes are also shown (no cMCSs with less than 3 cuts exist; the upper limit of cuts was set to 7).

The full/reduced networks contain 1668/564 metabolites and 2382/958 reactions (the reactions in the compressed network represent lumped reaction subsets). For computation 12 threads on a cluster node with two Intel Xeon DP X5650 processors (each 6 cores) were used.

As can be seen in Table 3, only a fraction (between 1.3% and 6%) of the computed MCSs up to size 7 turned out to be valid cMCSs. However, a large number of several thousand cMCSs could eventually be computed for each scenario.

We then analyzed the cMCSs in more detail. A first observation in Table 3 is that in three of the four scenarios considered cMCSs were found comprising only three reaction deletions; whereas for the case with smaller glucose uptake and higher demanded ethanol yield (scenario 2 in Table 3) at least 5 reaction removals are required. Generally, it is intuitive that expanding the space of undesired flux vectors in (22) and reducing the space of desired solutions in (23) by increasing 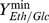 can lead to larger cMCSs since (i) a larger set of undesired flux vectors must be suppressed, and (ii) due to the reduced set of desired behaviors a smaller number of MCSs become admissible cMCSs. Hence, there is no cMCS in scenario 2 that is a subset of any cMCSs in scenario 1 in Table 3, but the other way around can occur. The same relationship exists between scenarios 3 and 4. Thus, the higher the yield that we want to guarantee by an intervention strategy, the larger is the required effort in terms of number of reaction knockouts.

**Figure 1:**
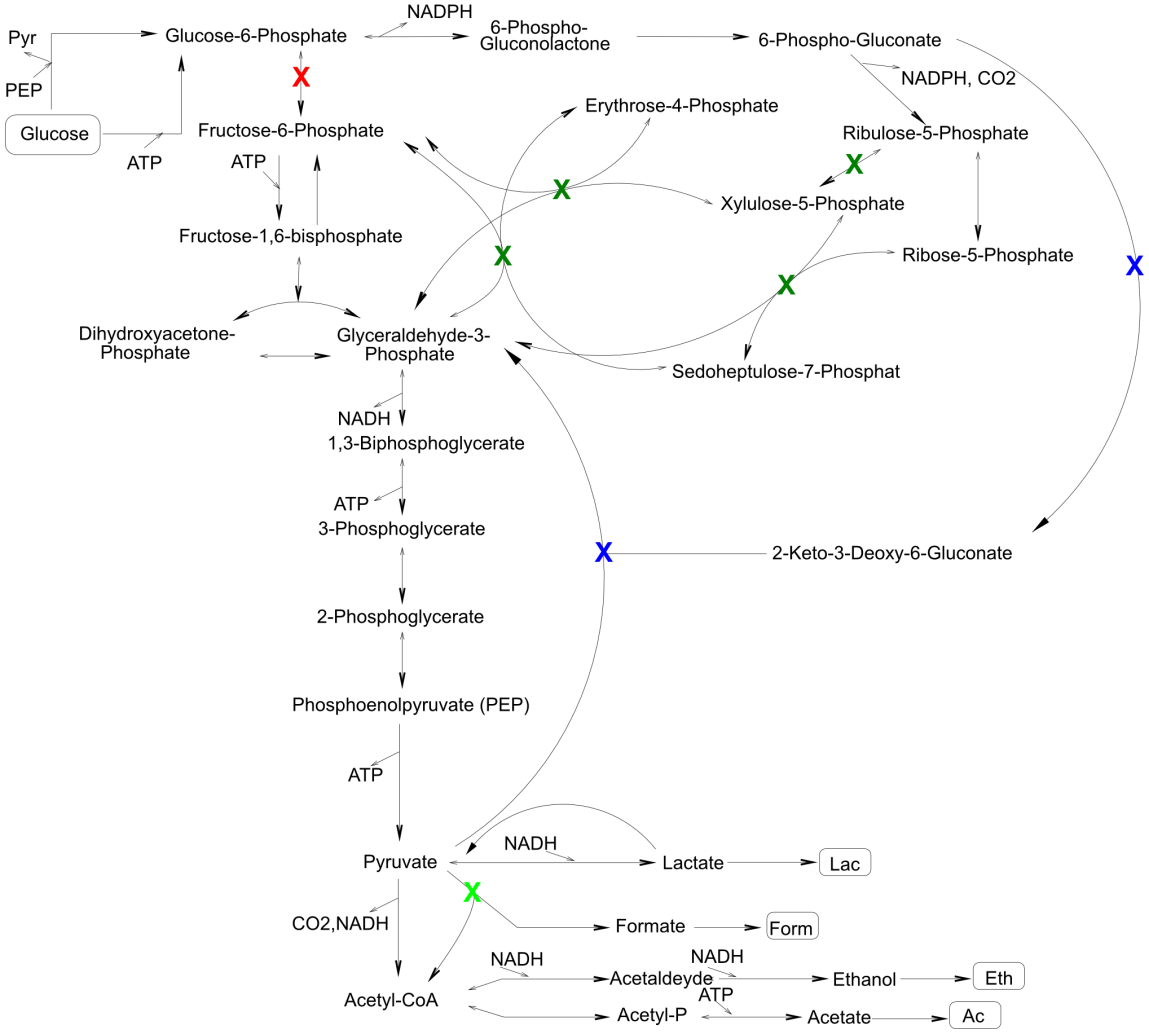
Constrained MCSs with three reaction knockouts leading to coupled ethanol and biomass formation by *E. coli* under anaerobic growth on glucose. The graphics indicates the found cMCSs requiring only three knockouts (Table 3). In total, 8 cMCSs were found for scenario 1 and two cMCSs for scenarios 3 and 4 (both being identical for the two scenarios). All these cMCSs cut the reaction with the red cross and one of the two reactions with a blue cross. In addition, for scenario 1, one of the dark green cuts has to be made whereas the two cMCSs for scenario 3 and 4 require the light green cut (see also explanations in the text).

The situation is different in the case of increasing 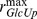. While the target flux polyhedron in (22) increases potentially demanding more cuts, the space of desired behaviors in (23) expands as well meaning that an MCS that was not a suitable constrained MCS in the case with smaller 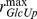 could now become a suitable cMCS. Hence, when increasing 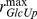, some cMCSs of a given size might disappear whereas others may arise as new solutions. This is also reflected by the cMCSs of size three which are depicted in Figure 1. All these cMCSs block central pathways for glucose degradation. An essential cut (red cross in Figure 1) for all cMCSs is that of the glucose-phosphate isomerase blocking upper glycolysis. In addition, all the considered cMCSs block the Entner-Doudoroff pathway by either cutting the phosphogluconate dehydratase or the 2-keto-3-deoxyphosphogluconate aldolase (blue crosses in Figure 1). In addition, for scenario 1 (with the smaller values for 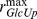 and 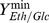), we have to cut one additional reaction out of 4 reactions of the pentose phosphate pathway (dark green crosses in Figure 1) whereas for scenarios 3 and 4 (whose two cMCSs of size three are identical) the third cut is given by the pyruvate-formate lyase reaction (light green cross in Figure 1). This result confirms that increasing 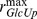 (from scenario 1 to scenario 3) may remove existing cMCSs but also produce new ones.

The cMCSs for scenario 1 (the red cut, one of the two blue cuts and one of the four dark green cuts in Figure 1) also illustrate the difference between reaction and enzyme/gene cut sets. Since two of the four reactions with a green cross are catalyzed by the same enzyme (transketolase) knocking out the corresponding two genes (there are two different transketolases in *E. coli*) would actually cut two reactions at the same time for which the model predicts that *E. coli* can not grow anymore. Thus, only four of the eight cMCSs remain valid on gene basis. However, as already explained earlier, those effects can be taken into account based on gene-enzyme-reaction associations.

The fact that three reaction or gene knockouts may suffice to induce a high ethanol yield of more than 1.8 (scenario 4) is a surprising fact on its own. Previous work on computing intervention strategies for ethanol overproduction in a smaller (core) network of *E. coli* showed that more than three reaction knockouts would be required to ensure a large ethanol yield (see, e.g., [10]). Given the results with three knockouts made herein, this might be a bit confusing since much more inefficient pathways will exist in a genome-scale network which must all be blocked. However, similar as discussed above for a scenario with increased substrate uptake rates, a larger network may also have additional high-yield metabolic routes (allowing coupled biomass and ethanol synthesis) not contained in the smaller network which could ‘survive’ a cut set for blocking the low-yield pathways. We can thus conclude that genome-scale network models may reveal metabolic engineering strategies that are smaller than those found in small- or medium-scale subnetworks. Importantly, one always has to keep in mind that an MCS predicts an intervention purely from stoichiometric relationships. Whereas blockage of the undesired flux vectors can be guaranteed if the network structure is correct, it can not ensure that the remaining pathways will have the capacity to carry a flux that is large enough to fulfil the requirements of the desired flux vectors. In addition, unknown regulatory constraints may further reduce the space of desired behaviors by which some cMCSs may become invalid.

We mention here that two other intervention strategies with three knockouts for production of ethanol by *E.coli* were presented in [9]. However, these solutions ensure high ethanol yield only if the cell grows at maximal growth rate whereas our interventions are more stringent since they guarantee a high ethanol yield for *any* growth rate of the mutant.

**Figure 2:**
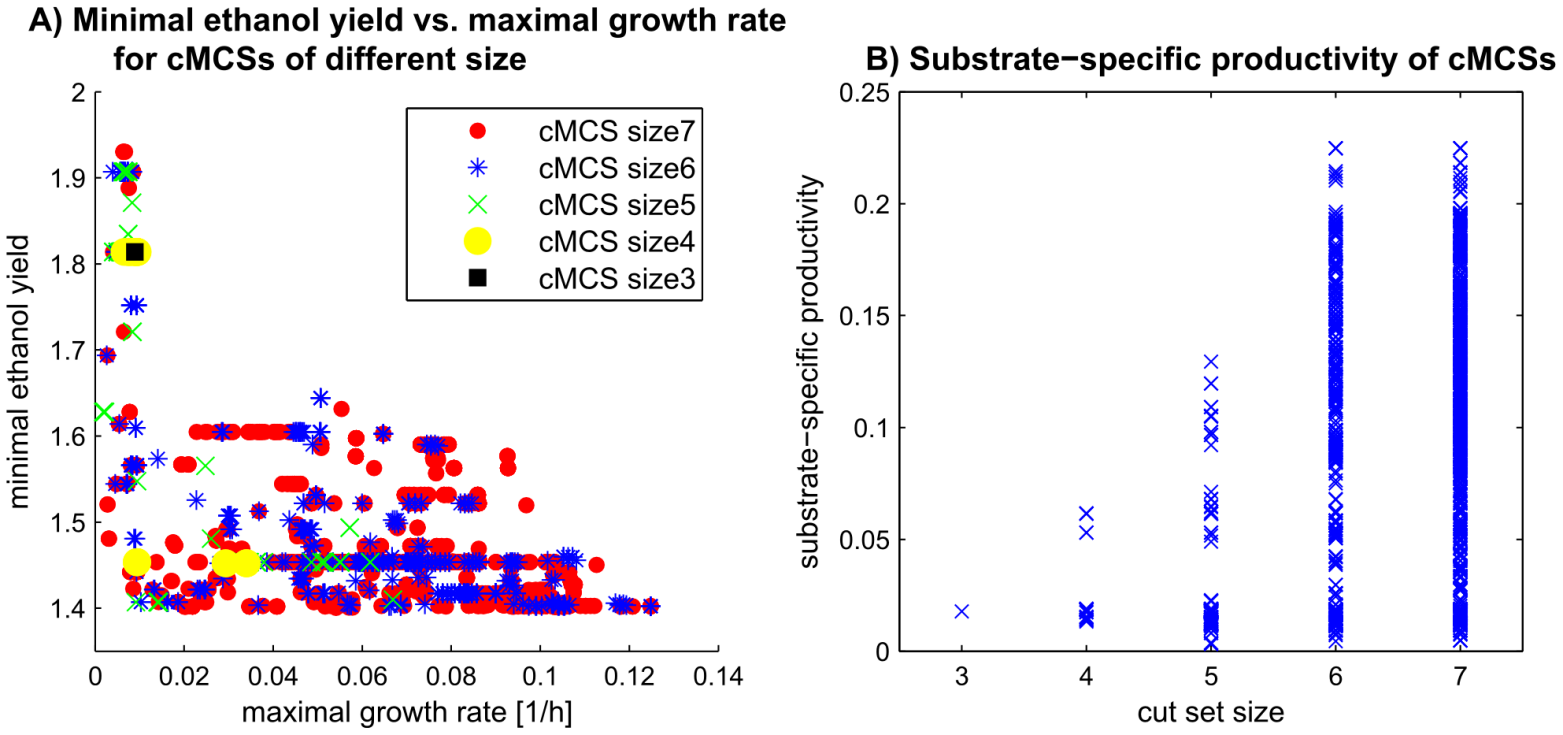
Performance measures of calculated strain designs for growth-coupled ethanol production in *E.coli*. A: Minimal (guaranteed) ethanol yield (under maximal substrate uptake rate) vs. maximal possible growth rate for each cMCS of scenario 3 in Table 3. The size of the cMCSs are indicated by different markers. It becomes apparent that when higher maximal growth rates are required larger cut sets become necessary implying also a decrease in the guaranteed ethanol yield. B: Substrate-specific productivity (SSP) induced by the cut sets of scenario 3 in Table 3. Cut sets were ordered with respect to the number of required knockouts. Note that some crosses represent several cMCSs having, for example, the same SSP.

Having exhaustively enumerated the cMCSs up to a given size enables one to analyze essential features and performance measures of all found intervention strategies by which eventually the optimal knockout strategy can be selected. Figure 2 shows exemplarily two such performance studies. Figure 2A displays for each cMCS of scenario 3 the relationship between (i) maximal growth rate, (ii) minimal (guaranteed) product yield (shown for maximal substrate uptake rate; the lower boundary for arbitrary substrate uptake rates still holds to be 1.4), and (iii) number of required reaction deletions (cut set size). It can be seen that most cMCSs (including those with the smallest size 3) achieve relatively low growth rates (lower than 0.1 *h^−1^*) and that in order to have a growth rate larger than 0.1* h^−1^* it is necessary to use cut sets with a least 6 knockouts. If higher growth rates and/or smaller cut sets are required the minimal product yield would have to be lowered.

Other performance measures of designed mutant strains can be studied as well. One such proposed measure is substrate-specific productivity (SSP) which is the product of the growth-rate and the product yield [29]. Figure 2B shows the SSP of all cMCSs computed for scenario 3. It can be seen that highest SSP values can only be achieved with cut sets of size 6 or 7. This illustrates again that a trade-off between number of knockouts and certain performance measures has sometimes to be made when eventually selecting an intervention strategy for implementation. Such a screen is greatly facilitated if all cut sets have been enumerated up to a certain size. More advanced screening methods for evaluating strain design strategies have been suggested in [30] and could readily be applied to calculated cMCSs.

As a technical note, it is not absolutely mandatory to have all MCSs (up to a maximal size) enumerated before running the LP checks for testing the “survival” of some desired flux vectors: these checks could be (independently) performed as soon as an MCSs has been found by the MILP solver. In fact, it is in principle possible to integrate the LP into the MILP so that the cMCSs are computed directly which offers the advantage that far fewer exclusion constraints need to be integrated while the enumeration proceeds. In practice, however, this approach showed a markedly inferior performance for the system studied here. One reason is that the LP adds further degrees of freedom to the solution space and leads to redundant solutions for the cMCSs which requires a more intricate control of the *populate* procedure to suppress these redundant solutions. Whether the integrated approach can be reformulated in a manner that facilitates a more efficient calculation of its cMCSs solutions is a potential topic for further investigation.

To summarize the results of this sub-problem, our algorithm enabled the enumeration of all reaction knock-out sets up to size 7 that lead to coupled ethanol and biomass synthesis in *E.coli*. To the best of our knowledge, this exceeds by far other attempts to enumerate such metabolic engineering strategies in large-scale networks.

If more computational capacity is available, one might try to find even larger cMCSs. However, the best knockout strategy to be implemented is likely to be contained among the up to 8819 smallest cMCSs found as the number of required interventions will be one (though not the only) key criterion when deciding for a concrete strain design.

### Constrained MCSs for coupling aerobic growth with fumarate or serine production in *E. coli*

One large-scale study to evaluate the growth-coupled production potential in *E.coli* has been presented by Feist *et al*. [29]. The aim was to identify strain designs based on reaction knockouts with a maximum production rate at optimal growth for a number of substrate/product pairs. This was achieved by first applying OptKnock [8] with a knockout limit of either three or five and then using the results in the initial population for OptGene which employs genetic programming as optimization method [31]. OptGene was then run with a time limit of one week to find additional strain designs with up to 10 knockouts. As underlying *E.coli* model the iAF1260 reconstruction [24] was taken and in order to reduce the search space the knockouts were restricted to a subset of about 150 reactions in the network. As minimum growth rate for the strains a limit of 0.1 *h^−1^* was chosen and an ATP maintenance of 8.39 *mmol/(gDW⋅h)* required. Both glucose and oxygen uptake were limited to 20 *mmol/(gDW⋅h)*. Given this setup it was possible to calculate strain designs for many substrate/product pairs but for some of them strains with only low productivity or even no strains with growth-coupled product synthesis were found.

Here we wanted to test the potential of our method for some of the intervention problems. We focused on the aerobic production of either fumarate or serine from glucose which both have a potential for high yield as calculated by FBA. However, growth-coupled strains for the production of fumarate only achieved 20% (5 knockouts, OptKnock) respectively 23% (7 knockouts, OptGene) of the theoretical maximum while for serine no growth-coupled strains could be identified in [29]. We therefore applied our approach to look for (additional) strain designs for these two configurations.

To demonstrate the power of our method in dealing with large-scale systems, we increased the search space drastically compared to [29] by allowing all reactions to be knocked out except for those that are either spontaneous or essential for the production condition. Since glucose is taken up under aerobic conditions, the same 152 reactions as for the calculation of the synthetic lethals above have also been removed. This results in 718 (fumarate) resp. 719 (serine) knockout candidates (compared to 150 candidates used in [29]). As the results in [29] suggested that growth coupling will be difficult for fumarate and serine production we chose a comparatively low minimal product yield of 0.5. This constraint together with the ATP maintenance requirement und the uptake limits was used to calculate MCSs that disable flux vectors with product yields below 0.5. Afterwards, only those (constrained) MCSs were kept that fulfil the minimal growth rate requirement.

For fumarate production, the MCSs up to size 7 were calculated (taking 13.6 h) from which 30 cMCSs (all of size 7) could be extracted. Applying those cMCSs would result in production strains exhibiting – at maximal substrate uptake rates – a guaranteed (minimal) fumarate yield between 0.71 and 0.89 corresponding to minimal production rates between 40.9% and 51.3% of the theoretical maximum of 34.68 *mmol/(gDW⋅h)* (note that the minimal yield for any substrate uptake rate is ensured to be 0.5 as demanded by the constraints for the desired flux vectors). As for the ethanol study, all these values are independent of the assumption of optimal growth. Likewise, in the case of serine production, the MCSs up to size 6 were calculated (taking 3.1 h) from which 140 cMCSs (all of size 6) could be extracted. These would result in strains with with a guaranteed serine yield between 0.71 and 0.91 (at maximal substrate uptake rate) corresponding to minimal production rates between 36.6% and 47.0% of the theoretical maximum (38.71 *mmol/(gDW⋅h)*). Hence, our results show that significantly larger fumarate production rates can be achieved with 7 knockouts than computed by OptGene. In case of serine where no suitable knockout strategy could be identified in [29], our method proves the existence of strain designs for coupled biomass and product synthesis and that 6 reaction knockouts would be theoretically sufficient to guarantee a serine yield of 47% of the theoretically maximal value. Moreover, tens of the smallest strain designs with 6 knockouts could be identified by our algorithm in a comparably fast way and larger ones could also be determined if desired.

## Discussion

In this work we presented MCSEnumerator, a new algorithmic approach to enumerate the smallest (c)MCSs up to a given size in genome-scale networks. This approach is based on a MILP problem calculating the shortest EMs in the dual representation of the metabolic network eventually yielding the smallest cMCSs. The whole procedure can be summarized by five steps:

1. Build the metabolic network as usual by specifying the stoichiometric matrix and the irreversibility constraints (equations (1) and (2)). Optionally, network compression steps can be applied.
2. Define the space of undesired (target) flux vectors and (optionally) the space of desired flux vectors by means of the linear inequalities (14) and (21), respectively. The (c)MCSs to be computed will ensure that no target flux vector can operate whereas the operation of at least one desired flux vector will be feasible.
3. Build the dual system which is immediately given by (17). Introduce indicator (or binary) variables (**z**) for the **v** variable and pose the MILP optimization problem for computing the shortest EM in the dual system (19).
4. Enumerate the *k*-shortest solutions (EMs) of the MILP problem from step 3 by using ALGO2.
5. Translate the EMs found in the dual to MCSs in the primal. If desired behaviors were specified in step 2, run one LP for each MCSs to check whether it is a constrained MCS, i.e., whether some desired flux distributions remain feasible after cutting the reactions contained in the MCS.

With these five steps, MCSEnumerator provides a generic approach for enumerating smallest intervention strategies; one just has to plugin the corresponding matrices in equation (17) and can then start the calculation using ALGO2.

Apart from the combination of dualization and shortest EM calculation in step 3, another key development made herein is the improvement of the required sub-routine for computing shortest EMs (ALGO2) which is now based on a more efficient enumeration of feasible EMs with fixed size and which consequently makes use of available enumeration features of modern MILP solvers. Appropriate integration of such functionalities could also be useful to effectively solve other enumeration problems in the field.

Despite the fact that calculation of *all* (c)MCSs with our approach is slower compared to other approaches requiring EMs to be calculated in a first step, it has the advantage that the smallest (c)MCSs, which are often the most interesting ones, can be found first and that no EMs need to be calculated beforehand. This property renders (c)MCSs calculation feasible in genome-scale networks. Also, the number of elements in an MCS has no major impact on the performance as it would have in brute-force enumerations (that exhaustively test all reaction subsets) and as it has been observed also for several directed search algorithms.

The main drawback of using a MILP stems from the fact that constraints have to be continuously added to remove already found MCSs and their supersets from the solution space. Hence this method is bound to slow down with increasing number of constraints which explains the inferior performance when computing *all* MCSs. However, the shown application examples demonstrated that our approach is capable to compute hundreds of thousands of smallest MCSs and several thousand smallest constrained MCSs in genome-scale networks (Table 3) which has not been achieved before. The large set of smallest cMCSs should suffice to characterize the space of the most efficient intervention strategies from which, in metabolic engineering applications, the most promising ones can be selected, possibly by screening the cMCSs via certain performance parameters.

The algorithmic advantage of the presented approach lies thus in the possibility to quickly (compared to other approaches) calculate the smallest (c)MCSs with neither network size nor the number of elements in the (c)MCSs posing major challenges. With these results and due to the fact that the approach of (c)MCSs allows the setup of complex intervention problems in a flexible and convenient way, we expect that a large number of metabolic network studies can benefit from our conceived framework.

An interesting aspect for future work will be to investigate how far ALGO2 (the sub-routine used for shortest EM calculation) can be generalized to enumerate also other elementary sets arising in different contexts of computational biology (e.g., for calculating minimal intervention sets in signaling or regulatory networks [32]).
